# S100A4 May Be a Good Prognostic Marker and a Therapeutic Target for Colon Cancer

**DOI:** 10.1155/2018/1828791

**Published:** 2018-07-09

**Authors:** Sabahattin Destek, Vahit Onur Gul

**Affiliations:** ^1^Bezmialem Vakıf University School of Medicine, Vatan Street, 34093 Fatih, Istanbul, Turkey; ^2^Department of General Surgery, Gulhane Education and Research Hospital, Ankara, Turkey

## Abstract

**Background:**

Globally, the colorectal cancers rank the third in terms of cancer incidence and rank the fourth in cancer-associated deaths. S100A4, an important member of the S100 protein family, serves to promote tumor progression and metastasis. By conducting this study, we aim to examine the role of S100A4 in the prognosis of colon cancer and to demonstrate its prognostic significance.

**Methods:**

Tissue samples of colon cancer from 148 patients who underwent colon resection due to colon cancer were analyzed by immunohistochemical staining to determine the protein expression levels of S100A4. The protein expression levels of S100A4 in tumor tissue were matched with the clinicopathologic factors including patient survival.

**Results:**

Cytoplasmic expression of S100A4 protein was demonstrated in the tumor tissue of 132 patients (89.2%) out of a total of 148 study patients. Statistically, the expression levels of the cytoplasmic S100A4 protein correlated significantly with the TNM stages and patient survival. The distribution of the S100A4 protein staining in the tumor tissue was associated with the age groups, tumor localization, TNM staging, and patient survival with statistical significance. The levels of S100A4 protein expression were found to be an independent prognostic factor for TNM staging and poor survival.

**Conclusion:**

Expression of the S100A4 protein in colon cancers may be an indicator of tumor progression and lymph node metastasis and may be useful for predicting the overall survival of the patients with colon cancer. In patients with colon cancer, it may be used as an indicator of poor prognosis.

## 1. İntroduction

 It is estimated that, in 2012, 14.1 million new cancer cases have emerged and 8.2 million deaths have occured due to cancer globally [1]. Lung (12.9%), breast (11.9%), and colorectal cancers (CRC) (9.7%) are the most common types of cancer. Among the most common causes of cancer-related deaths, colorectal cancers (8.5%) are in the fourth rank, preceded by lung, liver and stomach cancers [1].

CRC ranks the third in the USA, among all cancer types, with 10.3% incidence and 8.9% mortality rates [2]. On the other hand, it is in the second rank in Europe, among all cancer types, with 13% incidence and 12.2% mortality rates [3]. In our country, colorectal cancers are the third most common types of cancer (9%) according to the 2015 cancer statistics in Turkey [4].

Approximately 90% of cancer-related deaths are due to the metastatic dissemination of the primary tumors [5]. Tumor-node-metastasis (TNM) staging is an important prognostic parameter describing the depth of tumor invasion, lymph node involvement, metastasis status, and the stage of the tumor. Early diagnosis of tumors and metastases is critical to improving the treatment strategies and patient outcomes.

S100 proteins are dimeric, intracellular, and low-molecular-mass proteins binding Ca^2+^ [5]. S100A4 is found in the nucleus, cytoplasm, and the extracellular space, bearing a wide range of biological functions including angiogenesis, cell survival, motility, and regulation of intercellular adhesion [6]. High S100A4 expression levels are associated with aggressive tumor growth, metastases, and poor prognosis in colorectal cancers [5, 6]. In this study, we examined the association between the levels of S100A4 protein expression in the CRC tissue with the TNM stage.

## 2. Methods

A total of 148 patients, consisting of 89 males and 59 females, were included in the study. The hospital records were reviewed retrospectively and the following patient data were recorded including age, gender, tumor localization, type of the surgical intervention, tumor size, the grade of the tumor differentiation, and the presence of perivascular or perineural invasion; T, N, and M stages of the tumor; mortality and the duration of survival. S100A4 immunohistochemical staining was applied to the new slides prepared from the paraffin blocks and the levels of S100A4 protein expression were attained.

Patients with rectal cancer and patients having other types of tumors were excluded from the study. The patients with comorbid diseases were excluded from the study if these comorbidities led to the death of the patient during the follow-up period.

### 2.1. Immunohistochemistry

4-6 micrometer thick sections from the paraffin blocks of the tissue samples of the 148 patients were mounted onto the slides and were incubated at 37°C for 12 hours. After the sections to be stained with S100A4 were deparaffinized with xylene and high concentrations of alcohol, they were incubated in 3% hydrogen peroxide solution for 10 minutes to eliminate the endogenous peroxidase activity. For the antigen retrieval, they were boiled in a 6.0 pH-citric acid buffer for 10 minutes and then left for cooling at room temperature for 20 minutes. Following this step, 2-3 drops of Ultra V Block solution (LabVision, Fremont, USA) were dropped on the slides and they were left for 3-5 minutes. Then 1/100 diluted rabbit S100A4 antibody (DAKO, Carpinteria, CA, USA) was dropped onto the sections and they were incubated for 60 minutes at room temperature. After adding 2-3 drops of biotinylated rabbit antipolyvalent solution on the sections, they were left for 20 minutes. Between these steps, the sections were left in phosphate buffered saline washing solution for 3-5 minutes. Subsequently 2-3 drops of streptavidin-peroxidase solution were dropped on the sections and they were left for 20 minutes. After the sections were incubated in diaminobenzidine chromogen (Sigma, St Louis, MO, USA) for 5-10 minutes, they were stained with hematoxylin for 20 minutes for balancing. Alcohol was applied to the sections and they were dried in the drying oven.

### 2.2. Evaluation of the Immunohistochemical Staining

All sections subjected to immunohistochemistry were examined by two independent observers in a double-blinded fashion. Based on the diaminobenzidine chromogen brown, the valuation was performed examining the cytoplasmic staining for S100A4.

**Figure 1:**
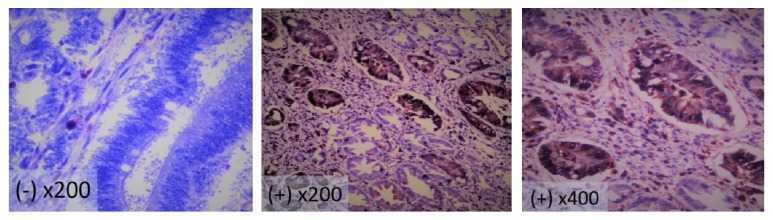
Expression of S100A4 in colonic mucosa ( - and + cytoplasmic staining ).

A section from a melanoma tissue sample was used as a positive control. Non-specific immune serum was used as the negative control. The tumor cells with brown-stained cytoplasms were classified as positive on the immunohistochemical evaluation of the sections. The S100A4 protein expression of the tumor cells was evaluated according to the ratio of the brown-stained tumor cells (Figure 1). In the presence of the tumor cells staining over 10%, it was accepted that the tumor expressed the S100A4 protein [5].

### 2.3. Statistical Evaluation

The data obtained in this study were analyzed by the IBM SPSS Statistics Version 20 software. Shapiro Wilks test was used to assess whether the variables were normally distributed. Relations between the groups of nominal variables were examined with the Chi-square analysis. Fisher's Exact Test and Pearson Chi-square analysis were applied when the volume of the expected values was insufficient. The significance level was accepted to be 0.05 while interpreting the results. A p value < 0.05 indicated a significant association between the variables, whereas p>0.05 indicated a nonsignificant association.

## 3. Results

Of 148 study patients, 89 (60%) were males and 59 (40%) were females. The age range of the patients was between 20 and 91 with a mean of 61.7 years. Of the study patients, 57% were 60 years old or older and 43% were younger than 60 years old. The majority of the patients were diagnosed with stage II colon cancers; more commonly having moderately differentiated adenocancers with diameters below 5 cm located in the right colon 89.19% of the patients had S100A4 expression in the tumor tissues revealed by immunohistochemical staining with a complete staining distribution of 52.03%. The tumor diameters ranged between 15 and 116 mm with a mean of 51.4 mm. Metastatic lymph nodes were identified in 72 (49%) patients. The number of the lymph nodes per patient ranged from 1 to 25 (mean 5). Distant organ metastases were present in 35 (24%) patients. The duration of survival ranged from 1 to 113 (mean 37.3) months.

As regards the association between the status of S100A4 expression and the clinicopathologic variables, no statistically significant associations were observed with the gender, age groups, tumor location, tumor size, tumor grade, the presence of vascular or neural invasions, mortality, or recurrence (p > 0.05). On the other hand, there was a statistically significant relationship between the S100A4 expression status and TNM stages (p <0.05). The primary stage was II in 56.25% of the patients without S100A4 expression and in 41.67% of the patients with S100A4 expression, whereas the primary stage was I in 31.25% of the patients without S100A4 protein expression and in 4.55% of the patients with S100A4 expression. There was a statistically significant association between the S100A4 expression status and duration of survival (p <0.05). The duration of survival was shorter than 2 years in 31.25% of the patients without S100A4 expression and in 71.97% of the patients with S100A4 expression. On the other hand, the duration of survival was in a range of 2-5 years in 18.75% of the patients without S100A4 protein expression and in 12.88% of the patients with S100A4 expression (Table 1).

A statistically significant association between the status of S100A4 staining in the tumor tissue and the age groups was observed (p <0.05). 45% of the patients with superficial staining, 33.3% of the patients with deeper staining, and 63.6% of the patients with complete staining were older than 60 years.

There was a statistically significant association between tumor tissue staining and the tumor localization (p <0.05). 57.5% of the patients with superficial staining, 33.3% of those with deeper staining, and 48.6% of those with complete staining had tumor localization in the right colon.

A statistically significant association between the status of staining in the tumor tissue and the TNM stages was present (p <0.05). 62.5% of the patients with superficial staining of the tumor tissues, 53.3% of the patients with deeper staining, and 28.6% of the patients with complete staining were diagnosed with TNM stage II tumors; however, 10% of the patients with superficial staining of the tumor tissues and 2.6% of the patients with complete staining were diagnosed with a primary stage of TNM I.

There was a statistically significant association between the status of tumor tissue staining and survival duration (p <0.05). 70% of the patients with superficial staining, 53.3% of the patients with deep staining, and 76.6% of the patients with complete staining had survival durations less than 2 years (Table 1).

No statistically significant associations of the tumor tissue staining status to gender, tumor grade, the presence of vascular or neural invasions, the diameter of the tumor, mortality, or recurrences were identified.

A statistically significant association between the tumor stages and S100A4 staging was present (p <0.05). S100A4 expression was identified in 63.6% of the patients at the T2 stage, in 81.3% of the patients at the T3 stage, and in 93.3% of the patients at the T4 stage.

A statistically significant association between the tumor stages and the status of staining in the tumor tissue was present (p <0.05). Of the patients with T2 tumors, 36.7% had superficial staining and 27.3% had complete staining of their tumor tissues. Of the patients with T3 tumors, 25% displayed superficial staining, 18.8% displayed deeper staining, and 37.5% displayed complete staining. Of the patients with T4 tumors, the staining was superficial in 26.8%, deeper in 10%, and complete in 56.7% (Table 2). There were no significant associations between the distribution of tumor staining and other clinicopathologic variables.

A statistically significant association between the nodal stages and S100A4 expression was present (p <0.05). S100A4 expression was identified in 80.26% of the patients with N0 disease, in 100% of the patients with N1 disease, and in 97.4% of the patients with N2 disease.

A statistically significant association between the nodal stages and the distribution of staining in the tumor tissue was present (p <0.05). Of the patients with N0 disease, 38.2% had superficial staining, 10.5% had deeper staining, and 31.6% had complete staining of their tumor tissues. Of the patients with N1 disease, 23.5% displayed superficial staining, 5.9% displayed deeper staining, and 70.6% displayed complete staining. Of the patients with N2 disease, the staining was superficial in 7.9%, deeper in 13.2%, and complete in 76.3% (Table 2).

There were no statistically significant associations of the metastasis stage to S100A4 expression or staining distribution in the tumor tissues.

## 4. Discussion

Colorectal cancer is one of the most common types of cancer especially in the developed countries and its incidence is on the rise. Approximately 1.4 million new colorectal cancer cases and 693,900 deaths due to CRC occurred in 2012. Increasing by 60% till 2030, these figures are predicted to reach 2.2 million new cases and 1.1 million deaths [7]. It is estimated that CRC will occur approximately in 5% of the global Western population [8]. The male/female ratio is 1.3 in colon cancer [1]. In our series, the male to female ratio was found to be 1.5. Colon cancers are more common between 60 and 75 years of age [2]. In our series, 57% of the patients were older than 60 years old. Colon cancer is most frequently found in the right colon with a rate of 25-30% [8]. In our series, half of the patients had right colon tumors.

The well-recognized prognostic factors for survival are the grade of the tumor, its depth of invasion, the presence of regional lymph node involvement, and distant metastases in CRC [9, 10]. Therefore, there is a need to develop reliable biomarkers and simple tests, which will be routinely applied for the early diagnosis of CRC, detection of its progression, determination of the prognosis, and the surveillance of the CRC patients [5, 10, 11].

S100 proteins were introduced for the first time in 1965 by Moore [12]. Found only in vertebrates, the S100 protein family consists of Ca^2+^ binding proteins at varying structures and sizes ranging from 9 to 13 kD. The number of the members of the S100 protein family has reached 25 currently [11, 13]. Their names originate from their solubility in 100% ammonium sulfate at the neutral pH [13]. S100 proteins are typically symmetrical dimers with each S100 subunit containing four *α*-helical segments. A major portion of these proteins contains a common calcium-binding motif called EF-hand [14, 15].

The S100 proteins do not display enzymatic activities. With the increased serum calcium levels, Ca^2+^ bound S100 proteins show their autocrine, paracrine, and systemic effects [13, 15]. S100 proteins interact with several target proteins intracellularly, including the enzymes, cytoskeletal structures like actin and myosin, several receptors, transcription factors, and nucleic acids, playing roles in homeostasis, energy metabolism, inflammation, migration, invasion, proliferation, differentiation, apoptosis, and intracellular Ca^2+^ regulation [13, 15].

Most of the members of the S100 family may take part in or initiate several biological functions contributing to malignancies, including proliferation, metastasis, angiogenesis, and protection from the immune response [12, 15]. S100 proteins may have specific activities on some target proteins such as NF-*κ*B, p53, and *β*-catenin [14, 15]. S100 proteins are expressed variably in various malignancies. Although their expressions decrease in some malignancies, they are usually increased [15]. The same S100 protein may be suppressive in a specific cancer type but may activate tumor generation in another type of cancer [15]. S100A4, which is a multifunctional Ca^2+^ signaling protein found in the cytoplasm and extracellular space, is also called metastasin (Mts1), pEL-98, 18A2, 42A, p9Ka, CAPL, calvasculin, and fibroblast-specific protein (FSP1) [11, 12, 16]. S100A4 was first described in 1984 and its expression was demonstrated first in 1989 [11, 17]. The human S100A4 gene is located on the chromosome 1q21 [15, 16]. The S100A4 expression has been reported in several cell types including fibroblasts, monocytes, macrophages, T-lymphocytes, neutrophilic granulocytes, and endothelial cells [6, 11].

The effect of S100A4 protein has been identified in several malignant, benign, or inflammatory diseases [12, 15]. The expression levels of S100A4 increase in rheumatoid arthritis, osteoarthritis, psoriasis, idiopathic inflammatory myopathies, inflammatory bowel diseases like Crohn's disease, cardiac hypertrophy, hepatic hemangiomas, and autoimmune diseases [6, 12, 18, 19]. S100A4 protein expression activates the proinflammatory processes mediated by tumor necrosis factor (TNF)-*α*, IL-1p and IL-6, and toll-like receptor (TLR)-4, augmenting the inflammation [12, 18, 19].

Several studies are available in the literature on the effects of S100A4 on tumor growth and metastases [12, 15]. Not only does S100A4 protein contribute to the expression levels during the course of aggressive disease processes but it also contributes directly to the progression of the disease as well [16]. S100A4 expression has been demonstrated in several malignancies such as malignancies of the pancreas, stomach, breasts, ovaries, kidneys, lungs, liver, prostate, and bones, in tumors of the urinary bladder, and in melanomas [6, 12, 15].

WNT-*β*-catenin signaling is one of the most important signaling pathways in the S100A4 protein associated colon carcinogenesis. Wnt proteins bind to the Frizzled receptors and coreceptors via the low-density lipoprotein receptor-related proteins (LRP) 5/6 and activate the receptor-specific signal flow, increasing the *β*-catenin in the cytoplasm. The increased *β*-catenin moves to the nucleus and forms a complex with the transcription factor 4 (TCF-4), initiating the S100A4 protein expression by transcription from the S100A4 target gene. The S100A4 protein expressed intracellularly moves to the extracellular space leading to the migration, invasion, and metastasis of the tumor cells [12–14, 20]. The adenomatous polyposis coli (APC) or mutations of the Smad4 gene found almost in 90% of the colorectal cases cause increased *β*-catenin levels in the cytoplasm. The accumulation of *β*-catenin in the nucleus causes tumor growth and leads to the expression of the genes taking part in tumor invasion [12, 16, 21].

The capacity for migration is a precondition for the cancer cell to enter the circulation. Matrix metalloproteinases (MMP) play important roles in this process. It is suggested that MMP is stimulated transcriptionally by the S100A4 protein, contributing to the angiogenesis and invasion of the tumor cells [6, 11, 16].

In the cell, the S100A4 protein interacts with the cytoskeletal proteins like nonmuscle myosin heavy chain (NMMHC) IIA, tropomyosin, actin, and filaments. S100A4 protein inhibits the activities of tropomyosin and NMMHC IIA in a Ca^2+^ dependent way binding calcium. Eventually, this increases the cellular mobility and migration [12, 14, 15]. The S100A4 protein interacts with liprin beta 1 and induces the invasiveness of the primary tumors [14]. S100A4 binds to the C-terminal of the p53 protein inhibiting the tumor suppressive effect of p53. This leads the tumor cell to act more aggressively [6, 12, 13].

The receptor for advanced glycation end products (RAGE) and annexin II mediates some of the extracellular functions of the S100A4 protein [6]. After being activated by the extracellular S100A4 protein binding, RAGE activates the mitogen-activated protein kinase (MAPK)/extracellular signal-regulated kinase (ERK) and NF-*κ*B signaling pathways, augmenting the potency for tumor growth, cell migration, and invasion in colon cancer [12, 15, 16].

The transformation process of the epithelial cells to the mesenchymal cell derivatives is called epithelial-mesenchymal transition (EMT). Induction of EMT leads to rapid tumor growth, invasion, and metastasis [12, 16, 22]. The target signaling protein for Wnt, the S100A4 gene expression, induces the EMT process, facilitating the progression of CRC and its potential for metastasis [14, 16, 22]. The protein expression of the gene encoding the LIM and SH3 domain protein (LASP1) enhances the S100A4 protein expression and leads to the activation of transforming growth factor *β* (TGF-*β*) through the activation of the Smad pathway. On the other hand, TGF-*β* facilitates the EMT induction and increases the levels of mitogenic growth factors leading to immunosuppressor and pro-angiogenic effects to occur [15, 16, 23].

The activation of the phosphatidylinositol 3-kinase (PI3K)/Akt/mTOR/p70S6K signaling pathway by the S100A4 protein causes migration, the expression of vascular endothelial growth factor, and E-cadherin downregulation [14, 16, 24]. When the E-cadherin expression is missing in the nucleus, the accumulated *β*-catenin in the cytoplasm increases the expression of S100A4, leading to the malignant EMT process and metastatic progression [6, 12, 16, 22].

Several studies demonstrated that various medications interacted with the S100A4 protein. Medications like cromolyn, amlexanox, phenothiazine, statins, propofol, arecoline, niclosamide, paquinimod, and sulindac decrease the activity of S100A4 protein [15, 16]. The antihelmintic medication niclosamide displays its anticancer effects by inhibiting the S100A4 promoter activity and by inhibiting the Wnt/*β*-catenin signaling pathway, which is a major regulatory pathway for the initiation of cancer, tumor growth, cell differentiation, and metastasis [15, 16]. The anti-inflammatory medication sulindac decreases the S100A4 activation and expression mediated by *β*-catenin, displaying antitumoral and anti-metastatic effects [6, 12, 15, 16].

Some chemotherapeutic medications may also be the treatment of choice as they have an impact on the S100A4 protein. It has been reported that paclitaxel can inhibit the S100A4 expression in the nucleus. Sorafenib can decrease the expressions of S100A4 mRNA and S100A4 protein. Calcimycin is a structural inhibitor of the active Wnt/*β*-catenin pathway signals and of the S100A4-promoter activity. By decreasing the expression of S100A4, it decreases the cellular motility and metastasis induced by S100A4 [12, 14–16].

The studies in the literature noted significant associations of the higher levels of S100A4 expression with the tumor localization, lymph node metastasis, TNM stages, and the depth of tumor invasion in patients with CRC [6, 11, 13]. On the other hand, it was reported that no significant associations of the higher levels of S100A4 expression existed with the age, gender, tumoral differentiation, the size of the tumor, the presence of vascular invasion, distant metastases, and presence of recurrences in CRC [11]. Unlike these findings, some studies observed increased rates of metastases during the surveillance of the patients with S100A4 expression [6, 15]. Higher S100A4 expression levels are associated with poor prognosis and lower rates of survival [11, 12, 20, 25].

In our study, too, an exaggerated S100A4 protein expression was demonstrated in 89% of all CRC patients. A statistically significant relationship was found between the S100A4 expression status and TNM stages. S100A4 protein expression is found in 54.5% of the TNM stage I patients: in 85.9% of the stage II patients, in 100% of the stage III patients, and in 94.2% of the stage IV patients. As the TNM stages advanced, the rate of the S100A4 protein expression increased. Parallel to the previous studies, our study did not demonstrate a significant association of the S100A4 expression with the age, gender, tumoral differentiation, the size of the tumor, the presence of vascular invasion, distant metastases, and presence of recurrences in CRC.

In our study, a statistically significant relationship was found between the S100A4 expression status and TNM stages in CRC. Similarly, a statistically significant relationship was found between the S100A4 expression status and the duration of survival. While the patients with S100A4 expression had a 34.5-month mean duration of survival, the mean duration of survival was 60 months in patients without S100A4 expression. S100A4 expression was demonstrated in 93% of the patients with a 5-year survival and in the 95% of the patients with a survival duration of 2 years or less. The survival parameters decreased along with the increased S100A4 expression.

The distribution of the S100A4 staining in the tumor tissue was assessed in order to understand the patterns of the superficial, deep, and complete staining. It was found that the S100A4 staining distribution was statistically associated with the age, localization of the tumor, TNM stages, and survival. In patients younger than 60 years old, a deeper staining pattern was observed. However, in patients older than 60 years old, a complete staining was displayed. In the right colon tumors, in TNM stage II patients, and in patients with a 2-year survival, the staining was complete.

As regards the statistically significant associations between the TNM stages and the levels of S100A4 protein expression, S100A4 expression was identified in 63.6% of the T2 stage patients, in 81.3% of the T3 patients, and in 93.3% of the T4 patients. As the stage of the tumor advanced, the rate of the S100A4 protein expression increased.

There was a statistically significant association between the TNM stages and staining distribution in the tumor tissues. 27.3% of the T2 patients, 37.5% of the T3 patients, and 56.7% of the T4 patients demonstrated complete staining. As the stage of the tumor advanced, the distribution of complete staining for S100A4 protein increased.

A statistically significant association between the TNM nodal stages and S100A4 expression was present. S100A4 expression was identified in 80.26% of the patients with N0 disease, in 100% of the patients with N1 disease, and in 97.4% of the patients with N2 disease. As the nodal stage of the tumor advanced, the rate of the S100A4 protein expression increased.

There was a statistically significant association between the TNM nodal stages and staining distribution in the tumor tissues. 31.6% of the N0 patients, 70.6% of the N1 patients, and 76.3% of the N2 patients had complete staining of their tumor tissues. As the nodal stage advanced, the distribution of complete staining for S100A4 protein increased. There were no statistically significant associations of the metastasis stage to S100A4 expression or staining distribution in the tumor tissues.

## 5. Conclusion

As our study has demonstrated, too, the exaggerated expression of the S100A4 protein indicates the capacity of the tumor for invasion and metastasis rather than the capacity for initiating a tumor formation. Our study has noted that as the T and N stages advanced, the exaggerated expression of S100A4 increased as well. The close association of the stage defining parameters with the S100A4 is significant in terms of prognosis. In this study of ours, it was noted that the S100A4 protein was a factor increasing the aggressiveness of the tumor and the occurrence of lymph node metastases. On the other hand, the facilitation of the S100A4 expression along with the advanced stages of the tumor is the most significant finding as the tumor staging is the most important factor in terms of the prognostic value. The S100A4 protein may be a beneficial marker to predict the carcinogenesis, tumor progression, and prognosis in colorectal cancers. We are of the opinion that this may affect the selection of the treatment regimens.

## Figures and Tables

**Table 1a:** (a) S100A4 expression.

**Clinicopathologic features**	**Variables**	**Absent**		**Present**		**Total**		**Chi-square Test**
		**n**	**%**	**n**	**%**	**n**	**%**	**p**
**TNM Stage**	**I**	5	31.3	6	4.6	11	7.4	**0.001**
	**II**	9	56.3	55	41.7	64	43.2	
	**III**	0	0	38	28.8	38	25.7	
	**IV**	2	12.5	33	25	35	23.7	

**Duration of Survival**	**< 2 years**	5	31.3	95	72	100	67.6	**0.003**
	**2-5 years**	3	18.3	17	12.9	20	13.5	
	**5-10 years**	8	50	20	15.2	28	18.9	

**Table 1b:** (b) Distribution of tumor tissue staining.

**Clinicopathologic features**	**Variables**	**Absent**		**Superficial**		**Deep**		**Complete**		**Total**		**Chi-square Test**
		**n**	**%**	**n**	**%**	**n**	**%**	**n**	**%**	**n**	**%**	**p**
**Age Groups**	**< 60 years **	4	25	22	55	10	66.7	28	36.4	64	43.2	**0.026**
	**> 60 years **	12	75	18	45	5	33.3	49	63.6	84	56.8	

**Colon-Tumor Localization**	**Right**	9	56.3	23	57.5	5	33.3	37	48.1	74	50	**0.003**
	**Transverse**	0	0	5	12.5	3	20	18	23.4	26	17.6	
	**Left **	4	25	7	17.5	1	6.7	10	13	22	14.9	
	**Sigmoid**	0	0	3	7.5	0	0	9	11.7	12	8.11	
	**Multicentric**	3	18.6	2	5	6	40	3	3.9	14	9.46	

**TNM Stage**	**I**	5	31.3	4	10	0	0	2	2.6	11	7.43	**0.001**
	**II**	9	56.3	25	62.5	8	53.3	22	28.6	64	43.2	
	**III**	0	0	4	10	3	20	31	40.3	38	25.7	
	**IV**	2	12.5	7	17.5	4	26.7	22	28.6	35	23.65	

**Duration of Survival**	**< 2 years**	5	31.3	28	70	8	53.3	59	76.6	100	67.6	**0.002**
	**2-5 years**	3	18.8	2	5	4	26.7	11	14.3	20	13.5	
	**5-10 years**	8	50	10	25	3	20	7	9.09	28	18.9	

**Table 2a:** (a) Tumor stage

**Pathologic features**	**Variables**	**T1**		**T2**		**T3**		**T4**		**Total**		**Chi-** **square ** **Test**
		**Number**	**%**	**Number**	**%**	**Number**	**%**	**Number**	**%**	**Number**	**%**	**p**
**S100A4 expression**	**Absent**	1	100	4	36.4	3	18.6	8	6.7	16	10.8	**0.001**
	**Present**	0	0	7	63.6	13	81.3	112	93.3	132	89.2	
	**Total**	1	100	11	10	16	100	120	100	148	100	

**Distribution Of Tumor Tissue Staining**	**Absent**	1	100	4	36.4	3	18.8	8	6.7	16	10.8	**0.007**
	**Superficial**	0	0	4	36.4	4	25	32	26.7	40	27	
	**Deep**	0	0	0	0	3	18.8	12	10	15	10.1	
	**Complete**	0	0	3	27.3	6	37.5	68	56.7	77	52	
	**Total**	1	100	11	100	16	100	120	100	148	100	

**Table 2b:** (b) Nodal stage

**Pathologic features **	**Variables**	**N0**		**N1**		**N2**		**Total**		**Chi-** **square ** **Test**
		**Number**	**%**	**Number**	**%**	**Number**	**%**	**Number**	**%**	**p**
**S100A4 expression**	**Absent**	15	19.7	0	0	1	2.6	16	10.8	**0.001**
	**Present**	61	80.3	34	100	37	97.4	132	89.2	
	**Total**	76	100	34	100	38	100	148	100	

**Distribution Of Tumor Tissue Staining**	**Absent**	15	19.7	0	0	1	2.6	16	10.8	**0.001**
	**Superficial**	29	38.2	8	23.5	3	7.9	40	27.0	
	**Deep**	8	10.5	2	5.9	5	13.2	15	10.1	
	**General**	24	31.6	24	70.6	29	76.3	77	52.0	
	**Total**	76	100	34	100	38	100	148	100	

## Data Availability

The [Statistical Study and Full Text Article] data used to support the findings of this study have been deposited in the [Harvard Dataverse] repository [https://doi.org/10.7910/DVN/EMCRNZ] (https://www.re3data.org/repository/r3d100010051). The [DATA TYPE] data used to support the findings of this study have been deposited in the [NAME] repository ([DOI or OTHER PERSISTENT IDENTIFIER]) (https://fairsharing.org/biodbcore-001080). The [DATA TYPE] data used to support the findings of this study are included within the supplementary information file(s).
